# Association of platelet function with depression and its treatment with sertraline in patients with chronic kidney disease: analysis of a randomized trial

**DOI:** 10.1186/s12882-019-1576-7

**Published:** 2019-10-29

**Authors:** Nishank Jain, Fei Wan, Monica Kothari, Anuoluwapo Adelodun, Jerry Ware, Ravi Sarode, S. Susan Hedayati

**Affiliations:** 1 Department of Internal Medicine, University of Arkansas for Medical Sciences, 5323 Harry Hines Blvd, MC 8516, Dallas, TX 75390 USA; 2 Department of Biostatistics, University of Arkansas for Medical Sciences, Little Rock, USA; 3 Department of Internal Medicine, University of Texas Southwestern Medical Center, 5959 Harry Hines Blvd, MC 8516, Dallas, TX 75390 USA; 4 Department of Physiology and Biophysics, University of Arkansas for Medical Sciences, Little Rock, USA; 5 Department of Pathology, University of Texas Southwestern Medical Center, Dallas, USA

**Keywords:** Depression, Chronic kidney disease, Platelet function, Platelet aggregation, Sertraline, Selective serotonin reuptake inhibitors

## Abstract

**Background:**

Major Depressive Disorder (MDD) can lead to adverse cardiovascular outcomes in patients with chronic kidney disease (CKD). Although one of the proposed mechanisms is heightened platelet activation, effects of MDD and its treatment with a selective serotonin reuptake inhibitor (SSRI) on platelet function in patients with CKD remain unclear.

**Methods:**

In a pre-specified analysis, changes from baseline to 12 weeks in whole blood platelet aggregation (WBPA) and plasma levels of E-selectin and P-selectin on treatment with sertraline vs. placebo were investigated in 175 patients with CKD (estimated glomerular filtration rate [eGFR] < 60 ml/min/1.73m^2^) and MDD (MDD+/CKD+) in a randomized, double-blind trial. Correlations between severity of depressive symptoms and platelet function were also analyzed. In order to investigate whether differences in platelet function were due to presence of CKD or MDD, we compared a subgroup of 49 MDD+/CKD+ patients with eGFR < 30 ml/min/1.73m^2^ to 43 non-depressed CKD controls (28 CKD with eGFR < 30 ml/min/1.73m^2^ [MDD−/CKD+] and 15 individuals with eGFR ≥90 ml/min/1.73m^2^ [MDD−/CKD-].

**Results:**

In MDD+/CKD+ individuals, there were no significant correlations between severity of depressive symptoms and platelet function, and no significant changes in platelet function after 12 weeks of treatment with sertraline vs. placebo. There were no significant differences in platelet function among MDD+/CKD+ patients and controls without MDD except in WBPA to 10 μM ADP (*P* = 0.03). WBPA to ADP was lower in the MDD−/CKD- group (8.0 Ω [5.0 Ω, 11.0 Ω]) as compared to the MDD−/CKD+ group (12.5 Ω [8.0 Ω, 14.5 Ω]), *P* = 0.01, and the MDD+/CKD+ group (11.0 Ω [8.0 Ω, 15.0 Ω]), *P* < 0.01.

**Conclusions:**

Heightened ADP-induced platelet aggregability was observed in CKD patients compared to controls with normal kidney function, regardless of presence of comorbid MDD, and treatment with sertraline did not affect platelet function. These findings suggest that increased platelet activation may not be a major contributory underlying mechanism by which depression may lead to worse cardiovascular outcomes in patients with CKD. Future studies should include positive MDD controls without CKD to confirm our findings.

**Trial registration:**

ClinicalTrials.gov identifier numbers: CAST Study: NCT00946998 (Recruitment Status: Completed. First Posted: July 27, 2009. Results First Posted: January 30, 2018). WiCKDonASA Study: NCT01768637 (Recruitment Status: Completed. First Posted: January 15, 2013. Results First Posted: April 19, 2019).

## Background

Chronic Kidney Disease (CKD) and Major Depressive Disorder (MDD) are independent risk factors for cardiovascular (CV) events, such as heart attack, stroke, and death [1–3]. MDD is more prevalent among patients with CKD as compared to the general population, such that nearly 25% of CKD patients are affected with MDD [4]. Heightened platelet reactivity [5, 6] and excessive stickiness of endothelial cells to platelets [7] are important potential mechanisms for this excessive CV risk, as supported by studies reporting elevated plasma levels of platelet and endothelial activation markers (i.e., beta-thromboglobulin [β-TG], platelet factor 4 [PF4], P-selectin and E-selectin) [8–17] and increased platelet aggregability in individuals with CKD [5], as well as in those with MDD [4, 14, 18]. Limited data also suggest that treatment of MDD with selective serotonin reuptake inhibitors (SSRIs) may reduce platelet aggregation and activation markers, primarily in individuals with coronary artery disease [15, 16, 19, 20].

Previous studies exploring association of depression severity with platelet activation involved patients with MDD alone and were limited by small sample sizes, lack of controls, and lack of comprehensive platelet function measurements using aggregometry in fresh blood [15–17, 19–22]. Data from these studies are also limited in establishing a consistent association between MDD presence and platelet activation [23]. To date, there are no studies to report effects of comorbid MDD on platelet function, or to analyze effects of treatment with SSRIs on changes from baseline in platelet function in patients with CKD.

In a *pre-specified aim* of the *Chronic Kidney Disease Antidepressant Trial* (CAST), we sought to determine whether 12 weeks of treatment with sertraline vs. placebo, in a randomized, double-blind trial, would result in changes from baseline in platelet aggregation and activation markers among patients with CKD and MDD (MDD+/CKD+), and whether serum levels of sertraline and its active metabolite, N-desmethylsertaline, at study exit correlated with platelet activation markers. We first investigated whether there was a correlation between severity of depressive symptoms at baseline, as measured by 16-item Quick Inventory for Depression Symptomatology Self Report (QIDS-SR_16_) scale, and levels of plasma platelet activation markers, E-selectin (CD62E) and P-selectin (CD62P), or increased whole blood platelet aggregation (WBPA) in MDD+/CKD+ patients. In order to investigate whether differences in platelet function were due to presence of CKD or MDD, a comparison was made between MDD+/CKD+ patients from the CAST cohort with non-depressed controls with and without CKD. In the following aims, we hypothesized that in patients with CKD and MDD: *Aim 1*. The severity of depressive symptoms at baseline would correlate positively with platelet function; *Aim 2*. 12-weeks of sertraline (vs. placebo) treatment would result in reduced levels of platelet activation markers and aggregation; *Aim 3*. Individuals with CKD and MDD would have increased platelet aggregability as compared with controls without MDD, regardless of kidney disease presence.

## Methods

### Study participants and design

For the first and second aims, we used 175 patients with *MDD and CKD* (**MDD+/CKD+**) from the Chronic Kidney Disease Antidepressant Sertraline Trial (CAST), a randomized, double-blind, placebo-controlled trial of sertraline in 201 adults with estimated glomerular filtration rate (eGFR) of < 60 ml/min/1.73, the methods and primary outcome results of which have been previously published and adhere to CONSORT guidelines [24, 25] Participants had eGFR of < 60 mL/min/1.73 m^2^ for at least 3 months, calculated using the 4-variable *Modification of Diet in Renal Diseases* (MDRD) equation. Kidney transplant recipients and those receiving renal replacement therapy were excluded. Detailed methods for the CAST study were published earlier [24, 25]. To be eligible, participants had to score ≥ 11 on the QIDS-SR_16_ at baseline and also meet criteria for a current MDD using the Diagnostics and Statistical Manual of Mental Disorders IV-based Mini Neuropsychiatric Interview (MINI) interview. Of the 201 participants, 175 underwent baseline measurements for platelet function at Visit 1 (randomization visit).

**Fig. 1:**
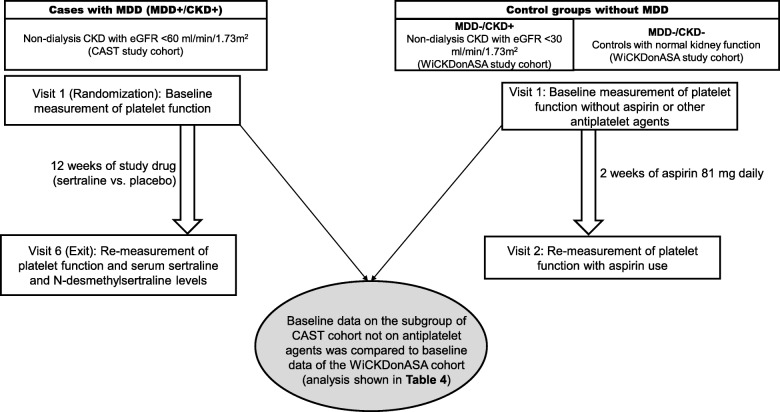
Participant Flow Diagram. The study design of the two trials from which the cohort was formed and time points for data collection. CKD, Chronic Kidney Disease; CAST, CKD Antidepressant Sertraline Trial; MDD, Major Depressive Disorder; WICKDonASA, Whole Blood Platelet Aggregation in Chronic Kidney Disease on Aspirin study

For the third aim, we used a subgroup of CAST participants (**MDD+/CKD+**) who had an eGFR < 30 ml/min/1.73m^2^ for comparison to sample of 43 controls *without MDD* from the *Whole Blood Platelet Aggregation in Chronic Kidney Disease on Aspirin* (WICKDonASA) study. The WICKDonASA study was a prospective controlled clinical trial of asymptomatic adults with and without CKD to evaluate platelet function in CKD individuals at baseline and in response to treatment with aspirin (Fig. 1) [5]. Controls without MDD included 15 individuals with normal kidney function and an eGFR of ≥90 mL/min/1.73 m^2^ (**MDD−/CKD-**) and 28 CKD individuals (**MDD−/CKD+**) with eGFR of < 30 mL/min/1.73 m^2^. MDD−/CKD- were included if there was no evidence of kidney disease as defined by an eGFR ≥90 mL/min/1.73 m^2^, urine albumin-to-creatinine (UACR) < 30 mg/g, and no detectable kidney disease by other methods as defined by the National Kidney Foundation guidelines. *MDD−/CKD+* controls were patients with CKD and an eGFR of < 30 mL/min/1.73 m^2^ for at least 3 months, calculated using the 4-variable MDRD equation. Kidney transplant recipients and those receiving renal replacement therapy were excluded. Absence of MDD in both groups was confirmed by a baseline QIDS-SR_16_ score < 11. We previously validated a score ≥ 11 to have the best diagnostic accuracy for presence of a MDD in CKD individuals, using the MINI interview as the gold standard [26]. Detailed inclusion and exclusion criteria for this study were previously published [5].

Institutional Review Board approval was obtained prior to the research procedures for both studies, and all participants provided written informed consent. All study procedures conformed to the Declaration of Helsinki. For both studies, participants were recruited from the same outpatient clinics at the University of Texas Southwestern (UTSW), Parkland Hospital, and Veterans Affairs North Texas Medical Centers in Dallas, Texas, from December 2012 to January 2014 (WiCKDonASA study) and March 2010 to November 2016 (CAST study) [5, 25].

### Study procedures and data collection

The study designs of the two trials, including time points for the collection of data used in the present study, are shown in the Fig. 1. For aims 1–2 involving CAST participants (MDD+/CKD+), eligible individuals who had MDD by the MINI interview and successfully completed a 1-week single-blind placebo run-in and still met the criterion of having a depressive symptom severity QIDS-SR_16_ score of ≥11 were randomized to sertraline 50 mg per day or matching placebo at visit 1. Blood and urine tests were also obtained on visit 1. Post-randomization, participants returned for regular follow-up of assessment of depressive symptoms and adverse events, and study drug dose titration. Drug dose was escalated by 50 mg daily every 2 weeks for a maximum tolerated dose of 200 mg daily during the first 6 weeks. The dose was kept constant for the final 6 weeks of the study period. Baseline whole blood platelet aggregation (WBPA) and plasma levels of E-selectin and P-selectin were obtained at visit 1, and stratified by baseline use of aspirin or other antiplatelet therapy (self-reported by the patient and confirmed using the electronic health records). At 12-weeks post randomization (exit), blood levels were re-measured for WBPA and platelet function markers, and serum sertraline and *N*-desmethysertraline levels (Fig. 1) quantified using high-performance liquid chromatography with fluorescence detection in the reference Mayo Medical Laboratories, Rochester, MN.

For the WiCKDonASA study, used for the MDD−/CKD+ and MDD−/CKD- controls, demographic, clinical, and medication use data were collected from the participant and confirmed using the electronic health records during the baseline visit (visit 1) when they were not receiving any treatment with aspirin or other antiplatelet agents. Blood tests drawn within a week prior to visit 1 were used for eGFR calculation and other baseline laboratory values. If such tests were not available, all baseline laboratory data were obtained via venipuncture at visit 1. Baseline values of WBPA and plasma E- and P-selectin were obtained from blood collected at visit 1, when participants were not on any antiplatelet therapy and prior to receiving prescription for aspirin, and repeated at visit 2 after 2 weeks of treatment with aspirin 81 mg daily (Fig. 1).

### Outcome measurements

The pre-specified primary outcome measure was platelet function, as measured by WBPA and platelet adenosine triphosphate (ATP) secretion induced by various agonists. Secondary outcome measures were plasma levels of platelet activation markers E-selectin (CD62E) and P-selectin (CD62P). In both the studies (CAST and WiCKDonASA), the same protocol was used for the collection of blood samples, platelet aggregometry, and enzyme linked immunosorbent assay (ELISA) procedures, and platelet function markers were measured in the same reference laboratory. Venipuncture was performed on fasting participants for complete blood count, measurement of WBPA and platelet activation markers by collecting whole blood in tubes containing 3.2% sodium citrate as the anti-coagulant (9:1 ratio). Blood was processed for platelet aggregation within 3 h of collection for all participants. WBPA induced by 0.25 and 0.5 mM arachidonic acid, 10 and 20 μM adenosine diphosphate (ADP), and 2 μg/mL collagen were measured using ex vivo whole blood impedance platelet aggregometry in a Chrono-log aggregometer (Chrono-log Corporation, Havertown, PA 19083, U.S.A.) at the UTSW Coagulation Laboratory, a Clinical Laboratory Improvement Amendments (CLIA) certified laboratory to perform aggregometry [27]. Using firefly luciferin-luciferase reaction in a photomultiplier tube (Chrono-log Corporation, Havertown, PA 19083, U.S.A.), platelet ATP secretion induced by various agonists including 1 U/mL thrombin were also measured. Whole blood impedance platelet aggregation is thought to be superior to plasma-based light transmittance platelet aggregation for assessing platelet function as it is more sensitive and faster, evaluates platelets in their physiological milieu in the presence of red blood cells (RBCs) and white blood cells (WBCs) known to affect platelet function, and does not require centrifugation which results in some platelet injury [28]. For the measurements of platelet activation markers, CD62P and CD62E, platelet poor plasma was obtained by centrifuging citrated whole blood at 4500 *g* for 20 min, frozen and stored at − 80 °C to run ELISAs in batches using kits from R&D Systems, Inc., Minneapolis, MN 55413, U.S.A., in the same laboratory at UTSW.

### Sample size considerations

Based on prior studies, mean (SD) WBPA to 0.5 mM arachidonic acid in healthy volunteers with normal kidney function *without* and *with* aspirin treatment was 12 ± 4 Ω and 1 ± 4 Ω, respectively [4]. In the pilot data on 63 participants with CKD and MDD from the CAST study, mean (SD) WBPA to 0.5 mM arachidonic acid *without* and *with* aspirin treatment were 8.3 ± 8.7 Ω and 5.6 ± 9.9 Ω, respectively. After log-transformation of this data, coefficient of variation was calculated to be 0.84 (SD/Mean 1.35/1.6 in log units). This coefficient of variation corresponded to being able to detect a 4 Ω clinically meaningful difference in geometric means of WBPA to 0.5 mM arachidonic acid between groups (CKD vs. controls with normal kidney function). With a sample size of 30 participants (20 CKD patients and 10 controls with normal kidney function), this study would have 84% power to detect a 4 Ω difference in WBPA between groups at a two-sided α of 0.05.

### Statistical analysis

Baseline characteristics were compared using one-way non-parametric analysis of variance (ANOVA) on the ranked outcome for continuous non-Gaussian variables, one-way ANOVA for continuous Gaussian variables, and Pearson Chi-square test for categorical variables between groups (sertraline vs. placebo). Correlation between severity of depressive symptoms, using the QIDS-SR_16_ score taken continuously, serum levels of sertraline and N-desmethylsertraline, and platelet function was tested using Spearman correlations. In a *pre-specified analysis of the CAST study*, Wilcoxon signed rank sum was used to compare platelet function at baseline and week 12 (exit) between treatment groups (sertraline vs. placebo). Analysis of covariance (ANCOVA) was used to assess differences in mean changes from baseline to 12 weeks between sertraline-treated vs. placebo-treated groups by testing whether the coefficient of treatment indictor was significantly different from zero using a t-test. The ANCOVA model included each platelet activation marker or component of WBPA at baseline and the binary treatment indicator (1 = sertraline, 0 = placebo). ANCOVA is a more robust approach than repeated measures models for skewed data [29, 30]. In subgroup analyses, baseline characteristics were compared using one-way non-parametric analysis of variance (ANOVA) on the ranked outcome for continuous non-Gaussian variables, one-way ANOVA for continuous Gaussian variables, and Pearson Chi-square test for categorical variables between groups (MDD+/CKD+, MDD−/CKD+, and MDD−/CKD-). Post hoc pairwise comparisons were performed among groups using two sample t-tests (continuous variables) and Fisher’s exact test (categorical variables) when the test statistics for overall differences were significant at a *p*-value < 0.05. Similar analyses were performed for comparing outcome measures between these groups. The analysis for this project was generated using SAS software, Version 9.4 of the SAS System for Windows (Copyright© 2012 SAS Institute Inc., Cary, NC, USA).

## Results

### Baseline characteristics

Of the 201 CAST participants with MDD+/CKD+ who were randomized, 193 participants with at least one post-randomization outcome assessment were included in the modified intention-to-treat analysis, with 97 being randomized to and receiving sertraline and 96, placebo [25]. Of those, 175 participants underwent baseline measurements for platelet function (WBPA and ATP secretion) and 90 for platelet activation (plasma levels of E-selectin and P-selectin). Of the 175 participants, 88 received sertraline and 87 placebo (Table 1). Mean (SD) age of the cohort was 58 (13) years. Twenty six percent were women, 55% African American, and 59% had diabetes mellitus. Mean (SD) eGFR of the cohort was 27 (13) ml/min/1.73m^2^. There were no differences in baseline characteristics between the sertraline and the placebo group (Table 1).

**Table 1:** Baseline Characteristics of Patients with Chronic Kidney Disease (CKD) and Major Depressive Disorder (MDD) Randomized to Sertraline versus Placebo

Variable	CKD+/MDD+ (*n* = 175)	Sertraline (*n* = 88)	Placebo (*n* = 87)	*P*-value
Age, years, mean ± SD	58 ± 13	58 ± 13	58 ± 13	0.95
BMI, kg/m^2^, mean ± SD	33.4 ± 17.2	31.4 ± 8.8	35.4 ± 22.7	0.13
Women, n (%)	46 (26)	24 (27)	22 (25)	0.77
African American, n (%)	97 (55)	50 (57)	47 (54)	0.78
Diabetes mellitus, n (%)	103 (59)	48 (55)	55 (63)	0.28
Proton pump inhibitor use, n (%)	46 (26)	21 (24)	25 (29)	0.46
Beta blocker use, n (%)	116 (66)	61 (69)	55 (63)	0.39
Statin use, n (%)	109 (62)	53 (60)	56 (64)	0.57
ACEI/ARB use, n (%)	102 (58)	53 (60)	49 (56)	0.60
eGFR, mL/min/1.73 m^2^, mean ± SD	27 ± 13	28 ± 13	27 ± 13	0.74
Urine albumin-to-creatinine ratio, mg/g, median (IQR)	337 (0.5–10,250)	312 (0.5–7032)	555 (9–7723)	0.19
Hemoglobin, g/dL, mean ± SD	12.4 ± 7.6	13.0 ± 10.4	11.9 ± 2.3	0.36
White blood cell count, K per μL, mean ± SD	7 ± 3	7.0 ± 2.8	7.0 ± 2.7	0.92
Platelet count, K per μL, mean ± SD	224 ± 72	223 ± 81	226 ± 61	0.80
Serum total cholesterol, md/dL, mean ± SD	169 ± 47	168 ± 45	169 ± 49	0.85
Serum triglycerides, mg/dL, mean ± SD	147 ± 88	139 ± 73	155 ± 102	0.25
Serum LDL, mg/dL, median (IQR)	86 (14–284)	89 (32–252)	85 (14–287)	0.88
Serum total bilirubin, mg/dL, mean ± SD	1.0 ± 5.6	1.4 ± 8.0	0.5 ± 0.2	0.28
Hemoglobin A1c, %, mean ± SD	7.0 ± 4.0	6.8 ± 1.7	7.3 ± 5.4	0.34
Serum albumin, g/dL, mean ± SD	3.8 ± 0.6	3.8 ± 0.6	3.8 ± 0.5	0.93
Serum uric acid, mg/dL, mean ± SD	8.3 ± 2.1	8.2 ± 2.4	8.3 ± 1.8	0.83
Serum calcium, mg/dL, mean ± SD	9.1 ± 0.9	9.1 ± 0.8	9.1 ± 1.0	0.62
Serum phosphorus, mg/dL, mean ± SD	4.1 ± 0.9	4.0 ± 0.8	4.2 ± 1.0	0.09
Serum parathyroid hormone, ng/L, median (IQR)	127 (2–1629)	125 (5–708)	137 (20–1629)	0.37
Serum 25-hydroxyvitamin D, pg/L, mean ± SD	28 ± 15	30 ± 16	26 ± 13	0.09
QIDS-SR_16_ score, median (IQR)	12 (10–21)	12 (3–21)	12 (10–18)	0.15

For Gaussian continuous variables, means ±SD and *P*-values for the one way ANOVA F-test are reported. For non-Gaussian continuous variables, median (minimum-maximum) and *P*-values for non-parametric Wilcoxon rank sum test are reported. For categorical variables, *P*-values for the Pearson Chi-Square test are reported

*Abbreviations*: *ACEI/ARB* Angiotensin converting enzyme inhibitor or angiotensin II receptor blocker, *BMI* Body mass index, *eGFR* Estimated glomerular filtration rate, *IQR* Interquartile range, *LDL* Low density lipoprotein, *SD* Standard deviation, *QIDS-SR*_*16*_–16-item Quick Inventory for Depression Symptomatology Self-Report. MDD+/CKD+ represents participants from the Chronic Kidney Disease Antidepressant Sertraline Trial (CAST) Study with major depressive disorder and chronic kidney disease

### Association between severity of depression, platelet function, and sertraline levels

There was a significant correlation between severity of depressive symptoms at baseline as measured by the QIDS-SR_16_ score and serum levels of sertraline and its active metabolite, N-desmethylsertraline, measured at exit after treatment in the MDD+/CKD+ group, *r* = 0.21 (*P* = 0.01) and 0.18 (*P* = 0.03), respectively (Table 2). However, there were no significant correlations between severity of depressive symptom scores and values of WBPA induced by various agonists at baseline (Table 2). Similarly, when the analysis was stratified based on use of aspirin or other antiplatelet therapy at baseline, no statistically significant correlations were observed between the severity of depression by QIDS-SR_16_ scores and either WBPA or P- and E-selectin levels in individuals treated or not treated with aspirin (*data not shown*).

**Table 2:** Correlations of Depression Severity at Baseline and Serum Sertraline and N-desmethylsertraline Levels at Exit with Platelet Function in Patients with Major Depressive Disorder and Chronic Kidney Disease

	Spearman correlation coefficient	*P*-value
Depression Severity at baseline by QIDS-SR_16_ Score^a^	*N* = 175	
WBPA to 0.5 mM arachidonic acid, Ω	− 0.05	0.55
WBPA to 0.25 mM arachidonic acid, Ω	− 0.06	0.46
WBPA to 20 μM ADP, Ω	− 0.16	0.06
WBPA to 10 μM ADP, Ω	− 0.11	0.16
WBPA to 2 μg/mL collagen, Ω	−0.10	0.18
ATP secretion to 0.5 mM arachidonic acid, nmoles	−0.04	0.63
ATP secretion to 0.25 mM arachidonic acid, nmoles	−0.02	0.79
ATP secretion to 2 μg/mL collagen, nmoles	−0.14	0.06
ATP secretion to 1 unit/mL thrombin, nmoles	−0.06	0.45
^b^Plasma P-selectin levels, pg/L	0.06	0.61
^b^Plasma E-selectin levels, pg/L	0.00953	0.93
^c^Serum Sertraline levels, pg/L	0.21	0.01
^c^Serum N-desmethylsertraline levels, pg/L	0.18	0.03
Serum Sertraline Levels in pg/L		
WBPA to 0.5 mM arachidonic acid, Ω	−0.0089	0.93
WBPA to 0.25 mM arachidonic acid, Ω	− 0.0650	0.51
WBPA to 20 μM ADP, Ω	−0.1092	0.35
WBPA to 10 μM ADP, Ω	0.0341	0.7
WBPA to 2 μg/mL collagen, Ω	−0.0154	0.88
ATP secretion to 0.5 mM arachidonic acid, nmoles	−0.08725	0.38
ATP secretion to 0.25 mM arachidonic acid, nmoles	− 0.1472	0.14
ATP secretion to 2 μg/mL collagen, nmoles	− 0.1513	0.12
ATP secretion to 1 unit/mL thrombin, nmoles	−0.2200	0.03
^b^Plasma P-selectin levels, pg/L	−0.0946	0.50
^b^Plasma E-selectin levels, pg/L	−0.2076	0.14
Serum N-desmethylsertraline levels in pg/L		
WBPA to 0.5 mM arachidonic acid, Ω	− 0.0045	0.96
WBPA to 0.25 mM arachidonic acid, Ω	− 0.0553	0.59
WBPA to 20 μM ADP, Ω	−0.1063	0.37
WBPA to 10 μM ADP, Ω	0.0510	0.60
WBPA to 2 μg/mL collagen, Ω	−0.0164	0.87
ATP secretion to 0.5 mM arachidonic acid, nmoles	−0.0789	0.43
ATP secretion to 0.25 mM arachidonic acid, nmoles	−0.1237	0.22
ATP secretion to 2 μg/mL collagen, nmoles	−0.1287	0.20
ATP secretion to 1 unit/mL thrombin, nmoles	−0.2012	0.04
^b^Plasma P-selectin levels, pg/L	−0.0554	0.40
^b^Plasma E-selectin levels, pg/L	−0.2166	0.12

*Abbreviations*: *ADP* Adenosine diphosphate, *ATP* Adenosine triphosphate, *QIDS-SR*_*16*_ 6-item Quick Inventory for Depression Symptomatology Self-Report, WBPA- whole blood platelet aggregation, Ω, ohms

^a^Baseline (visit 1) values were used for the QIDS- SR_16_

^b^Plasma samples available on 90 participants at randomization (Visit 1) and 50 participants at study exit (Visit 6) for P-selectin and E-selectin

^c^Serum samples available on 140 participants at study exit (Visit 6) for Sertraline and N-desmethylsertraline levels

### Effect of sertraline treatment on platelet function in MDD+/CKD+ group

There were no statistically significant differences in baseline values of WBPA, ATP secretion, or plasma levels of P-selectin and E-selectin between the sertraline vs. the placebo groups in CAST participants with MDD+/CKD+ (Table 3). Similarly, no differences were observed in platelet function at study exit between the two treatment groups, except for WBPA in response to 0.5 mM arachidonic acid (Table 3). However, as compared to the placebo arm, no differences were observed in changes from baseline to exit after 12-weeks of treatment with sertraline for arachidonic acid or the other platelet function markers (Table 3). Finally, there were no significant correlations between serum levels of sertraline or N-desmethylsertraline and WBPA or selectins at study exit, except for ATP secretion to 1 unit/mL of thrombin, Spearman correlation − 0.22 (*P* = 0.03) for sertraline and − 0.20 (*P* = 0.04) for N-desmethylsertraline (Table 2).

**Table 3:** Platelet Function Profile of Patients with Major Depressive Disorder and Chronic Kidney Disease at Baseline and After 12 Weeks of Sertraline Treatment

	Sertraline, *N* = 88			Placebo, *N* = 87			*P*-value		
Platelet function	Baseline	Week 12	Median change from baseline to week 12	Baseline	Week 12	Median change from baseline to week 12	Between-groups at baseline^a^	Between groups at Week 12^b^	Between groups for change^c^
A. Whole blood platelet aggregation induced by agonist (in ohms)									
0.5 mM arachidonic acid	5 (0, 20)	1 (0, 21)	−4.00	0 (0,10)	0 (0, 10)	0	0.08	0.04	0.14
0.25 mM arachidonic acid	0 (0, 17)	0 (0, 15)	0	0 (0, 14)	0 (0, 6)	0	0.40	0.22	0.07
20 μM ADP	10 (6, 14)	8 (5, 12)	−2.00	11 (8, 14)	11 (5, 15)	0	0.82	0.22	0.10
10 μM ADP	10 (5, 12)	10 (6, 14)	0	10 (7, 15)	10 (7, 15)	0	0.08	0.15	0.23
2 μg/mL collagen	20 (14, 26)	20 (15, 27)	0	21 (14, 30)	18 (13, 24)	−3.00	0.51	0.21	0.09
B. ATP secretion induced by agonist (in nmoles)									
0.5 mM arachidonic acid	0.35 (0, 0.76)	0.23 (0, 0.68)	−0.12	0.26 (0, 0.72)	0.14 (0, 0.52)	−0.12	0.50	0.27	0.16
0.25 mM arachidonic acid	0.20 (0, 0.67)	0.11 (0, 0.63)	−0.09	0.13 (0, 0.52)	0 (0, 0.30)	−0.13	0.42	0.12	0.17
2 μg/mL collagen	0.71 (0.43, 1.11)	0.62 (0.35, 0.88)	−0.09	0.52 (0.29, 0.91)	0.55 (0.33, 0.89)	0.03	0.05	0.74	0.13
1 unit/mL thrombin	1.17 (0.83, 1.73)	0.87 (0.63, 1.15)	−0.30	1.02 (0.73, 1.43)	0.90 (0.46, 1.37)	−0.12	0.09	0.71	0.39
C. Plasma levels of platelet activation markers (in pg/L)									
P-selectin	34.10 (23.47, 49.24)	32.99 (23.92, 42.73)	−1.11	36.36 (29.60, 41.20)	31.32 (27.41, 43.13)	−5.04	0.90	0.57	0.44
E-selectin	42.06 (30.30, 59.95)	40.35 (30.24, 60.30)	−1.71	43.53 (36.11, 51.33)	44.38 (35.81, 56.05)	0.85	0.97	0.41	0.34

Values are reported as median and interquartile range (IQR)

^a^Denotes Wilcoxon rank test *P*-values between groups (sertraline versus placebo) for baseline values

^b^Denotes Wilcoxon rank test *P*-values between groups (sertraline versus placebo) for week 12 (exit) values

^c^Denotes ANCOVA *P*-values for the group indicator; ANCOVA model included baseline value and group (sertraline versus placebo)

### Subgroup analysis

Of the 175 CAST study participants who had platelet function measured at baseline (Visit 1), 85 were not on any antiplatelet agents. Of those, 49 had CKD with eGFR < 30 ml/min/1.73m^2^. Forty-three participants (28 with MDD−/CKD+ and 15 MDD−/CKD- controls) in the WiCKDonASA study were included in the analysis as non-depressed controls. There were no differences in age, gender, and race between groups (MDD+/CKD+, MDD−/CKD+ and MDD−/CKD-) (Table 4). Participants with MDD+/CKD+ had higher depression severity scores as compared to controls without MDD (MDD−/CKD- or MDD−/CKD+) (Table 4). The MDD+/CKD+ group and MDD−/CKD+ group had comparable severity of kidney disease as evidenced by similar eGFR. Serum albumin, phosphorus, and parathyroid hormone levels were also similar between the MDD+/CKD+ and MDD−/CKD+ groups. However, urine albumin-to-creatinine ratio was higher in the MDD−/CKD+ group (Table 4).

**Table 4:** Comparison of Baseline Characteristics of Subgroup of Patients with Major Depressive Disorder (MDD) and Chronic Kidney Disease (CKD) with Controls without MDD

Variable	MDD+/CKD+ (*n* = 49)	MDD−/CKD+ (*n* = 28)	MDD−/CKD- (*n* = 15)	*P*-value
Age, years, mean ± SD	53 ± 12	52 ± 10	49 ± 11	0.55
BMI, kg/m^2^, mean ± SD	32.3 ± 9.9	32.0 ± 5.5	29.8 ± 4.9	0.56
Female, n (%)	20 (41)	16 (57)	8 (50)	0.38
African American, n (%)	27 (55)	15 (54)	4 (25)	0.14
Diabetes mellitus, n (%)	27 (55)	13 (46)	7 (44)	0.64
Proton pump inhibitor use, n (%)	10 (20)	1 (4)	1 (6)	0.07
Beta blocker use, n (%)	31 (63)	21 (75)	0 (0)	< 0.01
Statin use, n (%)	26 (53)	19 (68)	5 (31)	0.06
ACEI/ARB use, n (%)	24 (49)	12 (43)	5 (31)	0.46
eGFR, mL/min/1.73 m^2^, mean ± SD	22 ± 13^a^	17 ± 7^a^	101 ± 15^b^	< 0.01
Urine albumin-to-creatinine ratio, mg/g, median (IQR)	432 (27–4962)^a^	1282 (2–6469)^b^	5 (0–87)^c^	< 0.01
Hemoglobin, g/dL, mean ± SD	11.2 ± 2.1^a^	11.2 ± 1.7^a^	14.4 ± 1.7^b^	< 0.01
White blood cell count, K per μL, mean ± SD	7 ± 3	8 ± 2	6 ± 2	0.25
Platelet count, K per μL, mean ± SD	230 ± 80	232 ± 55	217 ± 55	0.77
Serum total cholesterol, mg/dL, mean ± SD	179 ± 54	170 ± 42	180 ± 33	0.72
Serum triglycerides, mg/dL, median (IQR)	131 (47–340)	124 (35–275)	112 (43–443)	0.85
Serum LDL, mg/dL, mean ± SD	92 ± 42	94 ± 38	102 ± 29	0.71
Serum total bilirubin, mg/dL, mean ± SD	0.5 ± 0.7	0.4 ± 0.2	0.7 ± 0.6	0.20
Hemoglobin A1c, %	6.4 ± 1.8	6.5 ± 1.5	6.2 ± 0.8	0.80
Serum albumin, g/dL, mean ± SD	3.7 ± 0.6	4.0 ± 0.5	4.2 ± 0.4	< 0.01
Serum uric acid, mg/dL, mean ± SD	7.7 ± 2.0	8.9 ± 2.0	5.1 ± 1.8	< 0.01
Serum calcium, mg/dL, mean ± SD	8.9 ± 0.9	9.1 ± 0.8	9.3 ± 0.3	0.11
Serum phosphorus, mg/dL, mean ± SD	4.6 ± 1.0^a^	4.6 ± 1.1^a^	3.3 ± 0.3^b^	< 0.01
Serum parathyroid hormone, pg/mL, median (IQR)	184 (11–708)^a^	170 (77–255)^a^	42 (27–82)^b^	< 0.01
Serum 25-hydroxyvitamin D, ng/mL, mean ± SD	27 ± 17	28 ± 14	29 ± 15	< 0.01
QIDS-SR_16_ score, median (IQR)	12 (5–19)^a^	4 (0–8)^b^	3 (0–9)^b^	< 0.01

Abbreviations: *ACEI/ARB* Angiotensin converting enzyme inhibitor or angiotensin II receptor blocker, *BMI* Body mass index, *eGFR* Estimated glomerular filtration rate, *IQR* Interquartile range, *LDL* Low density lipoprotein, *SD* Standard deviation, *QIDS-SR*_*16*_ 16-item Quick Inventory for Depression Symptomatology Self-Report

For Gaussian continuous variables, means ±SD and *P*-values for the one way ANOVA F-test are reported. For non-Gaussian continuous variables, medians (minimum and maximum) and *P*-values for one way non-parametric ANOVA for the ranked outcomes F-test are reported. For categorical variables, *P*-values for the Pearson Chi-square test are reported. Pairwise comparisons using two sample t-test or Fisher’s exact test are reported when the overall F-test is significant; pairs with statistical differences have been designated with different alphabetical superscripts

MDD+/CKD+ represents subgroup of the Chronic Kidney Disease Antidepressant Sertraline Trial (CAST) Study cohort with major depressive disorder plus chronic kidney disease with entry eGFR of < 30 ml/min/1.73m^2^. MDD−/CKD+ represents controls with chronic kidney disease with entry eGFR of < 30 ml/min/1.73m^2^ without major depressive disorder from the Whole Blood Platelet Aggregation in Chronic Kidney Disease on Aspirin (WiCKDonASA) study. MDD−/CKD- represents controls without major depressive disorder or chronic kidney disease from the WiCKDonASA study

**Fig. 2:**
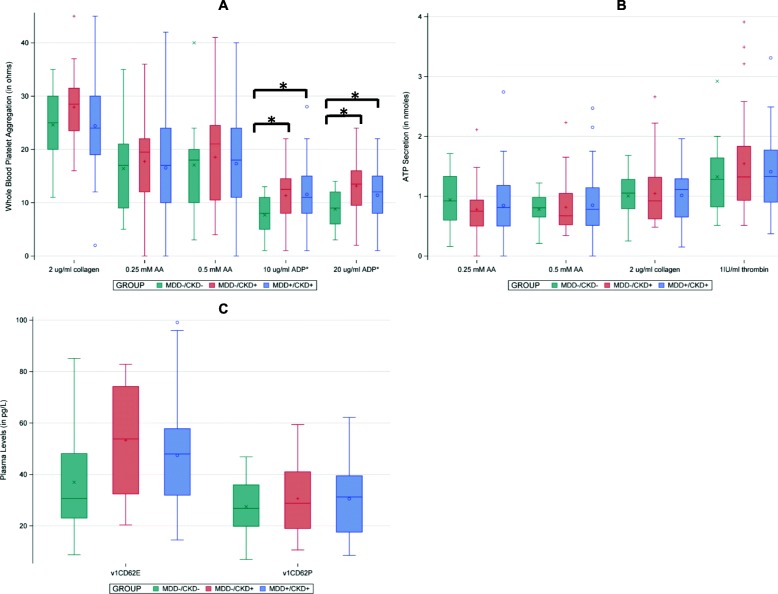
Grouped Boxplot Diagram. To investigate whether differences in platelet function were due to presence of chronic kidney disease (CKD) or major depressive disorder (MDD), comparisons were made between 49 patients with MDD and CKD (MDD+/CKD+), 15 non-depressed controls with normal kidney function (MDD−/CKD-) and 28 non-depressed controls with CKD (MDD−/CKD+) using non-parametric ANOVA on ranked outcomes. Panel **a** Represents comparison of whole blood platelet aggregation induced by agonists including collagen, arachidonic acid (AA), adenosine diphosphate (ADP). Overall *P* = 0.03 for difference between 3 groups for WBPA induced by 10 μM ADP and *P* = 0.02 for 20 μM of ADP; Panel **b** Represents comparison of platelet ATP secretion induced by agonists including collagen, arachidonic acid (AA), and thrombin; and, Panel **c** Represents comparison of plasma levels of P-selectin (CD62P) and E-selectin (CD62E). Asterisks represent significant *p*-values in pairwise comparisons

There were no statistically significant differences between baseline (visit 1) WBPA induced by various agonists except for ADP (Fig. 2a, b, and c). WBPA induced by ADP was statistically significantly different among the three groups when 10 μM of ADP was used as the agonist, *P* = 0.03 (Fig. 2a). In pairwise comparisons, the MDD−/CKD- group had lower WBPA to 10 μM ADP (8.0 Ω [5.0 Ω, 11.0 Ω]) as compared to the MDD−/CKD+ group (12.5 Ω [8.0 Ω, 14.5 Ω]), *P* = 0.01, and to the MDD+/CKD+ group (11.0 Ω [8.0 Ω, 15.0 Ω]), *P* < 0.01 (Fig. 2). In pairwise comparisons, the MDD−/CKD- group also had a lower WBPA to 20 μM ADP (9.0 Ω [6.0 Ω, 12.0 Ω]) as compared to the MDD−/CKD+ group (13.5 Ω [9.5 Ω, 16.0 Ω]), *P* = 0.01, and to the MDD+/CKD+ group (12.0 Ω [8.0 Ω, 15.0 Ω]), *P* = 0.01 (Fig. 2a).

## Discussion

The new finding in this study is that treatment with an SSRI, sertraline, vs. placebo in a randomized, double-blind, controlled trial did not result in changes from baseline in platelet function in individuals with CKD and MDD. Other than a small negative correlation with ATP secretion to thrombin, there were no significant correlations observed between serum levels of sertraline and N-desmethysertraline and platelet function. In addition, we did not find a significant correlation between platelet function and severity of depressive symptoms in patients with CKD. Finally, we found heightened ADP-induced platelet aggregation in patients with CKD compared to controls with normal kidney function, which did not increase incrementally with presence of comorbid MDD.

First, we report no correlation between severity of depressive symptoms (measured by QIDS-SR_16_ scores) and platelet function in patients with CKD and MDD. To our knowledge, there are no other published studies investigating this correlation in CKD samples with MDD. Depression increases the risk of adverse cardiac outcomes in patients with CKD and in those with underlying cardiovascular disease, such as acute coronary syndrome (ACS) [21]. Altered platelet activation due to altered serotonin metabolism in MDD is thought to be one of possible underlying mechanisms that has not been fully substantiated by published clinical evidence. Serotonin does not activate platelets per se according to in vitro studies, but potentiates platelet activation at low concentrations [31]. Therefore, it is plausible that severity of depressive symptoms may not directly correlate with platelet function. In addition, it is possible that changes in platelet activation observed in the setting of clinical depression may not be a major contributory mechanism by which depression may lead to worse cardiovascular outcomes in patients with CKD. The correlation between severity of depressive symptoms and serum levels of sertraline (and N-desmethylsertraline) observed at study exit in the CAST study participants was likely due to the measurement-based care protocol where participants with persistent and higher levels of depressive symptoms received escalation in the dose of the study drug (sertraline vs. placebo) [24].

Second, we did not observe a sertraline effect on plasma markers of platelet activation or platelet aggregation after 12 weeks of treatment in CKD patients with depression, except for a small negative correlation between sertraline and N-desmethylsertraline serum levels and ATP secretion in response to thrombin. In vitro studies analyzing effects of another SSRI medication, citalopram, on isolated platelets reported minimal inhibition of platelet aggregation induced by thrombin, collagen and ADP [19, 20]. In a non-randomized clinical trial of 19 MDD patients compared to 20 healthy controls, 24-weeks of therapy with escitalopram did not change platelet aggregability or clot firmness [16]. A subgroup analysis of the Sertraline Antidepressant Heart Attack Trial (SADHART) was the first randomized, placebo-controlled trial that reported 24 weeks of treatment with sertraline, vs. placebo, was associated with a decrease in serum markers of platelet activation (P-selectin and β-TG) in 64 patients with MDD and acute coronary syndrome [21]. Another subgroup analysis of the same study in 26 patients demonstrated negative correlations between plasma levels of sertraline and N-desmethylsertraline and platelet activation markers [32]. In our study, we not only measured circulating plasma levels of P-selectin and E-selectin, but also performed a comprehensive panel of whole blood platelet aggregometry and ATP secretion to various agonists before and after sertraline treatment in a CLIA-certified coagulation reference laboratory. Our study adds to the findings of previous reports in a larger sample size using randomization and blinding to report no major differences in platelet function and aggregation in patients with CKD and MDD after 12 weeks of sertraline therapy. Importantly, it is the first study to report treatment effects of an SSRI on platelet function in a CKD sample.

Third, this is the first study reporting increased ADP-induced whole blood platelet aggregation at baseline in patients with CKD independent of the presence of MDD. To our knowledge, this is the only controlled study published to date analyzing comprehensive platelet function (platelet aggregation and ATP secretion) and platelet activation markers (CD62P and CD62E) of CKD patients with depression. Previous studies reporting platelet activation in MDD were conducted in patients with normal kidney function. Studies using small samples generally reported increased platelet aggregability and activation markers in patients with depression but without CKD. In a study of 21 depressed patients (diagnosed using QIDS-SR_16_ and MINI interview) and 25 non-depressed controls, levels of circulating markers of platelet activation including levels of PF4, β-TG, P-selectins and E-selectins were analyzed from frozen serum samples [22]. This study reported depressive symptoms, as measured by QIDS-SR_16_ score, was associated with elevated plasma levels of platelet activation markers [22]. In a more recent study of 19 patients with depression and 20 controls without depression, platelets from depressed patients demonstrated elevated mean platelet volumes (*p* < 0.01), significantly increased aggregation induced by arachidonic acid and augmented expression of platelet surface receptors such as glycoprotein 1b (GPIb) by flow cytometry (*p* < 0.05) [16]. Another study of 22 treatment naïve MDD patients and 27 healthy controls without CKD measured platelet aggregation in plasma by light transmittance optical platelet aggregation. This study reported increased ADP-induced platelet aggregation in the group with MDD when 5 μM ADP was used as the agonist [17]. However, these studies were limited by small sample sizes, failure to perform comprehensive platelet function testing that included platelet aggregation and ATP secretion to various agonists, or use of plasma-based light transmittance optical platelet aggregation [16, 17, 22]. Our study used a large sample of patients with MDD and CKD (*N* = 175) and non-depressed controls with and without CKD (*N* = 43). In addition, we used whole blood without centrifuging blood samples to test platelet function in the physiological milieu in the presence of WBCs and RBCs. Therefore, our results address limitations of previous studies, and suggest that effects of depression as a comorbid condition on platelet function may not be as profound in CKD patients as in the general population. As such, serotonin (5-HT) was reported to potentiate only low concentrations of ADP-induced platelet aggregation [31], and hence, we may not have been able to detect a very small MDD-induced incremental increase in platelet aggregability in patients with an underlying ADP defect due to CKD.

We previously reported that CKD patients have increased ADP-induced platelet aggregation compared to controls with normal kidney function [5]. Therefore, these results extend the findings from our previous work. However, there are no clinical or in vitro studies to explore mechanisms by which the presence of advanced kidney disease, such as with eGFR of < 30 ml/min/1.73m^2^, may result in increased ADP-induced platelet aggregation, and whether this phenomenon is a result of increased expression of P2Y12 receptor on platelet surface or its increased downstream signaling in individuals with this underlying chronic disease.

This study has several strengths including a large sample size; using the same protocol for the collection of blood samples for platelet aggregation and a single CLIA-certified reference coagulation laboratory to standardize ascertainment of the outcome variables in all 3 cohorts; randomization and blinding in the CAST study to ascertain sertraline treatment effects on platelet function; and, finally, inclusion of two comparison groups as negative controls without MDD (CKD individuals and controls with normal kidney function) to discriminate between the differences in platelet function due to depression vs. due to CKD. Despite these strengths, potential limitations include a lack of measurement of platelet surface protein expression using flow cytometry to better ascertain platelet activation and, perhaps, the lack of a positive control group with MDD but without CKD. The available trial data did not include a positive MDD control without CKD (MDD+/CKD-) to clearly ascertain whether there was any MDD effect on platelet reactivity independent of CKD presence. Lack of power should always be considered as a possible explanation for the inability to detect significant differences between groups. However, our study, with a larger sample size than previously reported, should have had adequate power to detect a difference between groups where previous studies were able to detect a statistically significant difference with smaller sample sizes [15–17, 19–22]. Our sample size considerations suggest that the study was adequately powered to detect a difference for this analysis.

## Conclusions

In conclusion, depressive symptoms did not correlate with platelet aggregability or function, and treatment of MDD with sertraline did not change platelet function in patients with CKD and MDD. In addition, heightened ADP-induced platelet aggregability was observed in CKD patients as compared to controls with normal kidney function, regardless of presence of comorbid MDD. These findings suggest that increased platelet activation may not be the major contributory underlying mechanism by which depression may lead to worse cardiovascular outcomes in patients with CKD. Future studies should include positive MDD controls without CKD to confirm our findings.

## Data Availability

The datasets used and/or analyzed during the current study are available from the corresponding author on reasonable request.

## Abbreviations

ACS: Acute coronary syndrome
ADP: Adenosine diphosphate
ANCOVA: Analysis of covariance
ANOVA: Analysis of variance
ATP: Adenosine triphosphate
BMI: Body mass index
CAST: Chronic kidney disease antidepressant sertraline trial
CKD: Chronic kidney disease
CLIA: Clinical Laboratory Improvement Amendments
CV: Cardiovascular
eGFR: Estimated glomerular filtration rate
ELISA: Enzyme linked immunosorbent assay
LDL: Low density lipoprotein
MDD: Major depressive disorder
MDRD: Modification of diet in renal diseases
MINI: Diagnostics and Statistical Manual of Mental Disorders IV-based Mini Neuropsychiatric Interview
PF4: Platelet factor 4
QIDS-SR_16_: 16-item Quick Inventory for Depression Symptomatology Self Report
RBC: Red blood cell
SADHART: Sertraline Antidepressant Heart Attack Trial
SSRI: Selective serotonin reuptake inhibitor
UACR: Urine albumin-to-creatinine ratio
UTSW: University of Texas Southwestern Medical Center, Dallas, Texas, U.S.A.
WBC: White blood cell
WBPA: Whole blood platelet aggregation
WiCKDonASA: Whole blood platelet aggregation in chronic kidney disease on aspirin study
βTG: Beta-thromboglobulin
